# A London Pharmacist's Forecast

**Published:** 1912-06-08

**Authors:** 


### DISPENSERS UNDER THE INSURANCE ACT.

### A London Pharmacist's Forecast.

Some misunderstanding appears to exist concerning the position of dispensers under the National Insurance Act, and as that measure will come into operation soon a few further explanations will be useful. We therefore have pleasure in publishing a forecast of the dispensers' position after the Act comes into force from a London pharmacist of standing :—

"The opinion has been freely expressed that dispensers will be worse off than they are at present, but there is no ground for pessimism; as a matter of fact, the position of dispensers, especially of those who have passed no examination, will be considerably improved. Dispensers are, of course, divided into three classes; in one are those who have passed the qualifying examination of the Pharmaceutical Society, in another dispensers under the Apothecaries Act, and in the third those who, while they hold the diploma of no examining authority, have had practical experience in dispensing. The first-mentioned class will not only be able to dispense medicines for insurance patients; they will be among the privileged few who will be eligible to make 'arrangements' with local committees to dispense medicines—that is to say, they will be able to go on the chemists' panel, and some of them may find it worth while to open pharmacies, since there will be a vast increase in the amount of dispensing in chemists' shops.

Until the regulations of the Insurance Commissioners are published, it may be assumed that except in remote country districts medical practitioners will not be allowed to supply medicines to insured persons, and there is no sound reason to suppose that the Commissioners will deem it expedient to make regulations contrary to the explicitly stated intention of the Act. The effect of the wholesale transfer of dispensing from the medical man to the pharmacist will undoubtedly be to diminish the number of dispensers employed by practitioners, but those who are so displaced need have no anxiety, for pharmacists will require more assistants, and will be anxious to secure the services of those who are already experienced. The Act requires that pharmacists who make arrangements with Insurance Committees for the dispensing of medicines shall 'undertake that all medicines supplied by them to insured persons shall be dispensed either by or under the direct supervision of a registered pharmacist or by a person who, for three years immediately prior to the passing of the Act, has acted as a dispenser to a duly qualified medical practitioner or a public institution.'

"Thus for the first time dispensers whose only qualification is experience are given a statutory position—a very important fact, which has been overlooked by many. The privilege, it should be noted, extends only to those who have dispensed for a medical practitioner or a public institution; it is not shared by those who have dispensed for a pharmacist, and obviously pharmacists will desire to obtain the assistance of experienced dispensers who can dispense medicines without being under their direct supervision.

"In a short time it will not be too early for such dispensers to offer their services in this connection, and there is no reason why they should expect a salary lower than that which they have been accustomed to receive. The great majority of dispensers—perhaps practically all—qualified under the Apothecaries Act will come under this category, but, apart from this fact, there is nothing in the Act to affect the position of apothecaries' assistants. Moreover, it must not be forgotten that the Pharmaceutical Society has given an undertaking to promote a Bill to provide for a qualification for dispensers, and in doing so to seek the support of the General Medical Council, the Apothecaries Society, and the War Office, so as to satisfy existing interests. It should be clear that dispensers have no cause for anxiety."

